# Identification of novel ubiquitin receptors on the 26S proteasome by photo-crosslinking mass spectrometry

**DOI:** 10.1016/j.jbc.2026.111481

**Published:** 2026-04-22

**Authors:** Nicole S. MacRae, Ken C. Dong, Hiromitsu Harimoto, Andreas Martin

**Affiliations:** 1 Department of Molecular and Cell Biology, University of California at Berkeley, Berkeley, California, USA; 2 California Institute for Quantitative Biosciences, University of California at Berkeley, Berkeley, California, USA; 3 Biophysics Graduate Group, University of California at Berkeley, Berkeley, California, USA; 4 Howard Hughes Medical Institute, University of California at Berkeley, Berkeley, California, USA

**Keywords:** ATPases associated with diverse cellular activities (AAA), proteasome, proteostasis, protein degradation, ubiquitin

## Abstract

The 26S proteasome is the endpoint of the ubiquitin-proteasome system, an essential pathway for maintaining cellular homeostasis through targeted degradation of misfolded, damaged, and obsolete proteins. Substrates labeled with ubiquitin are directed to the 26S proteasome by binding to one or more ubiquitin receptors. However, ubiquitin-dependent degradation occurs even when the canonical receptor sites are mutated, suggesting the presence of additional, unidentified binding sites. Here we created photo-crosslinkable probes for ubiquitin interactions by incorporating the unnatural amino acid p-benzoyl-L-phenylalanine into ubiquitin. We show that these probes can be used to measure apparent affinities for known receptors and to reveal novel ubiquitin-binding sites on the yeast 26S proteasome. Through photo-crosslinking mass-spectrometry experiments, we identified a groove on the top of the proteasome, formed by Rpn2, Rpn9, Rpn10, and Rpn12, that serves as an additional tubiquitin-binding interface. Our photo-crosslinkable probes thus serve as versatile tools for the characterization of ubiquitin-protein interactions and the identification of ubiquitin-binding domains.

The ubiquitin-proteasome system is responsible for the degradation of damaged and obsolete proteins, and therefore plays essential roles in maintaining cellular homeostasis and the regulation of numerous vital processes (1, 2). Dysfunction of the ubiquitin-proteasome system can cause protein aggregation, neurodegenerative diseases, and various cancers. Most proteins are targeted to the 26S proteasome through the enzymatic attachment of ubiquitin to one or more of its lysine residues, with additional ubiquitins then added to lysines in ubiquitin itself to form poly-ubiquitin chains of different linkage types (3). The 26S proteasome consists of the 20S core particle (CP), which is a barrel shaped compartmental protease. One or both sides of the 20S CP are capped by the 19S regulatory particle (RP), which recruits ubiquitinated substrates, deubiquitinates them, and uses ATP hydrolysis for their mechanical unfolding and translocation into CP for proteolytic cleavage (4, 5). The 20S core consists of four stacked hetero-heptameric rings: two outer rings formed by the subunits α1-α7 (SCL1, PRE8, PRE9, PRE6, PUP2, PRE5, and PRE10 in yeast *Saccharomyces cerevisiae*), and two inner rings with the subunits β1-β7 (PRE3, PUP1, PUP3, PRE1, PRE2, PRE7 and PRE4 in yeast), of which β1, β2, and β5 have proteolytic activity. The 19S RP can be further split into two subcomplexes, the base and the lid. The base contains the heterohexameric AAA + ATPase motor formed by Rpt1-Rpt6, the large scaffolding subunit Rpn2, and two characterized ubiquitin receptors, Rpn1 and Rpn13 (6, 7, 8, 9). The lid consists of eight scaffolding proteins, Rpn3, 5, 6, 7, 8, 9, 12, and Sem1, and the essential deubiquitinase Rpn11. A third, integral Ub receptor, Rpn10, bridges the base and lid in the assembled 19S RP (10, 11). To be suited for proteasomal turnover, substrates need to contain a bipartite degradation signal consisting of polyubiquitin or multiple monoubiquitin modifications and an unstructured initiation region for engagement by the ATPase motor (12). Upon ubiquitin binding to one or more receptors, the initiation region enters the central channel of the ATPase hexamer. Engagement of the substrate polypeptide by the ATPase subunits triggers a major conformational change of the 19S RP from a resting state (s1) to processing states (non-s1), in which the ATPase subunits then use a hand-over-hand mechanism to drive the mechanical substrate threading through the central channel and into the 20S CP (5).

Ubiquitin interactions with the three canonical receptors present on the 19S RP, Rpn10, Rpn13, and Rpn1, were previously characterized by NMR spectroscopy, isothermal calorimetry (ITC), and surface plasmon resonance (SPR) (6, 7, 13, 14, 15). These studies identified specific residues responsible for the unique ubiquitin-binding behaviors of these receptors. Rpn10 binds ubiquitin through residues 228 to 232 in its C-terminal ubiquitin-interacting motif (UIM), while using its N-terminal von Willebrand factor type A (VWA) domain to interact with subunits of the 19S RP (13, 15). Rpn13 is bound to Rpn2 at the top of the proteasome and interacts with ubiquitin through the S2-S3, S4-S5, and S6-S7 loops of its pleckstrin-like receptor for ubiquitin (Pru) domain (7, 14, 16). Rpn1 binds to ubiquitin and the ubiquitin-like (UBL) domain of the cofactor Rad23 through its T1 site, and utilizes its T2 site to interact with the UBL domain of the deubiquitinase Ubp6 (6). It has also been reported that an N-terminal fragment of Rpn1, called the N1 site (Rpn1^214-335^), serves as an additional receptor for ubiquitin and UBL domains (17). All three receptors thereby contact a common interface on ubiquitin, the I44 patch (18), yet additional interactions confer some ubiquitin-linkage specificities, with Rpn1 and Rpn10 showing higher affinities for K48-linked ubiquitin chains, whereas Rpn13 seems to be less selective for this linkage type (19).

Interestingly, while mutation of all three ubiquitin receptors in *S. cerevisiae* causes decreased rates of ubiquitin-dependent degradation and increased canavanine sensitivity, the triple mutant is not fatal and does not completely eliminate ubiquitin-dependent substrate degradation by the proteasome, suggesting that there are additional, unidentified, or cryptic receptors (6). Although the identification and characterization of ubiquitin-binding proteins has been enabled by pulldowns, NMR, and ITC, we aimed to develop a more generic tool for probing ubiquitin receptors and identifying novel binding interfaces on ubiquitin-interacting proteins and complexes, such as the proteasome.

To build a probe for ubiquitin receptors, we sought to incorporate a photo-crosslinkable unnatural amino acid in key positions within ubiquitin. We chose p-benzoyl-L-phenylalanine (BPA), which reacts with C−H bonds and allows crosslinking to any amino acid within a ∼ 3 Å radius when excited by 365 nm light (20, 21). Upon irradiation, BPA forms a triplet benzhydryl diradical that can exist up to 120 μs and abstract a hydrogen radical from a nearby C−H bond, leading to a recombination and formation of a stable C−C bond (22). Because BPA’s diradical activation is reversible, it can be repeatedly activated over an extended time to obtain reasonable yields of crosslinking despite its relatively low reactivity. However, this repeated activation may also lead to trapping of nonspecific interactions. BPA has previously been used to capture protein interactions (23), including with ubiquitin (24, 25, 26, 27), and for substrate crosslinking to the proteasome (28). Here, we incorporated BPA at four different sites in ubiquitin to develop photo-crosslinkable probes for the identification and characterization of ubiquitin-binding domains (UBDs). Due to BPA’s rather low crosslinking efficiency, we were able to reasonably recapitulate the ubiquitin-binding affinities of canonical proteasome receptors that were previously determined by other biochemical methods, highlighting the potential of these probes for characterizing ubiquitin−UBD interactions in general. We then used these probes to identify novel ubiquitin-binding interfaces on the 26S proteasome and found a groove formed by Rpn2, Rpn9, Rpn10, and Rpn12 on top of the proteasome, which may be used for multivalent binding of longer or branched ubiquitin chains during substrate delivery.

## Results

### Design and development of photo-crosslinkable ubiquitin probes

**Figure 1:**
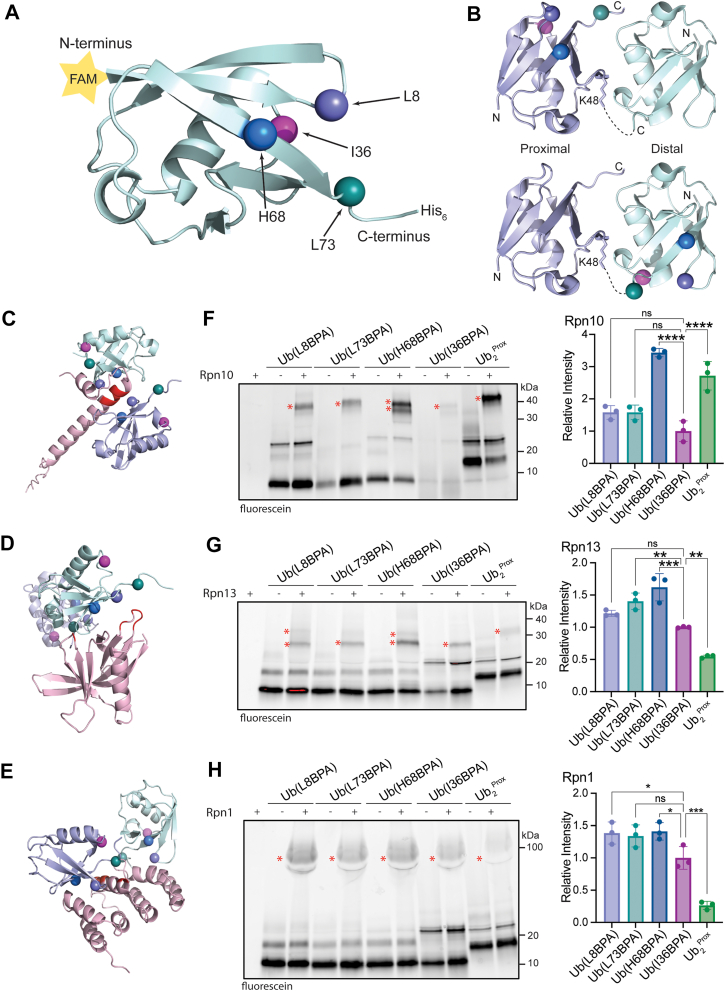
**Development of photo-crosslinkable ubiquitin probes.***A*, structure of ubiquitin (PDB: 1UBQ) with positions of BPA incorporation indicated by spheres and the N-terminal FAM label represented by a yellow star. *B*, BPA incorporation into proximal and distal ubiquitin moieties of a K48-linked di-ubiquitin. Shown is the crystal structure of di-ubiquitin (PDB: 7S6O) with the BPA locations indicated by spheres. *C*, AlphaFold model of Ub_2_ (proximal Ub in *purple*, distal Ub in *light blue*) bound to the UIM of yeast Rpn10 (*pink*). The residues of the UIM responsible for Ub binding (L228, A229, M230, A231, and L232) are shown in *red* and the BPA positions in Ub_2_ are shown by *spheres*. *D*, structure (PDB: 6UYI) of Ub_2_ (*purple* and *light blue*) bound to human Rpn13 (*pink*). The residues responsible for Ub binding (in yeast, E41, E42, L43, F45, and S93) are highlighted in *red*. *E*, structure (PDB: 2N3V) of Ub_2_ (*purple* and *light blue*) bound to the T1 site of yeast Rpn1 (the corresponding fragment of Rpn1 is shown in *pink*). Residues responsible for Ub binding (D541, D548, and E552) are highlighted in *red*. *F*, *left*: gel analyzing the crosslinking of FAM-Ub(L8BPA), FAM-Ub(L73PA), FAM-Ub(H68BPA), FAM-Ub(I36BPA), and K48-linked FAM-Ub(H68BPA)-Ub(K48 R) (Ub_2_^Prox^) with Rpn10. Crosslink bands are marked with ∗. *Right*: quantification of crosslink bands, relative to the total intensity of crosslinked and uncrosslinked species and normalized to FAM-Ub(I36BPA). Shown are the mean values and standard deviations of the mean for three technical replicates. Statistical significance was calculated using a one-way ANOVA test: ∗∗∗∗*p* < 0.0001; ns, *p* > 0.05. *G*, *left*: gel analyzing the crosslinking of FAM-Ub(L8BPA), FAM-Ub(L73PA), FAM-Ub(H68BPA), FAM-Ub(I36BPA), and K48-linked FAM-Ub(H68BPA)-Ub(K48R) (Ub_2_^Prox^) with Rpn13. Crosslink bands are marked with ∗. *Right*: quantification of crosslink bands, relative to the total intensity of crosslinked and uncrosslinked species and normalized to FAM-Ub(I36BPA). Shown are the mean values and standard deviations of the mean for three technical replicates. Statistical significance was calculated using a one-way ANOVA test: ∗∗∗*p* < 0.001;∗∗*p* < 0.01; ns, *p* > 0.05. *H*, *left*: gel analyzing the crosslinking of FAM-Ub(L8BPA), FAM-Ub(L73PA), FAM-Ub(H68BPA), FAM-Ub(I36BPA), and K48-linked FAM-Ub(H68BPA)-Ub(K48R) (Ub_2_^Prox^) with Rpn1. Crosslink bands are marked with ∗. *Right*: quantification of crosslink bands, relative to the total intensity of crosslinked and uncrosslinked species and normalized to FAM-Ub(I36BPA). Shown are the mean values and standard deviations of the mean for three technical replicates. Statistical significance was calculated using a one-way ANOVA test: ∗∗∗*p* < 0.001; ∗*p* < 0.05; ns, *p* > 0.05. PDB, Protein Data Bank; BPA, p-benzoyl-L-phenylalanine; FAM, fluorescein amidite; Ub2, ubiquitin dimers; UIM, ubiquitin-interacting motif.

To develop probes for ubiquitin interactions, we used genetic code expansion to incorporate the unnatural amino acid BPA into four ubiquitin constructs, which also contained an N-terminal GlyGly extension for fluorescein conjugation by a sortase reaction and a C-terminal His_6_-tag for purification and pulldowns (Fig. 1*A*). We used the orthogonal aminoacyl-tRNA synthetase (MjTyrRS)/tRNA pair that leads to the incorporation of BPA at any position in the coding sequence where an amber stop codon was introduced (21, 28) and thus allows the site-specific placement of the crosslinker in ubiquitin. We designed three probes containing BPA in or near the canonical hydrophobic I44 patch on ubiquitin, Ub-(L8BPA), Ub(H68BPA), and Ub(L73BPA), as well as one probe with BPA more distant from this interface, Ub(I36BPA), to test for the degree of nonspecific crosslinking. All probes also contained an N-terminal fluorescein amidite (FAM) label. As many ubiquitin receptors specifically bind chains of ubiquitin, we designed two K48-linked ubiquitin dimers (Ub_2_): Ub_2_^Prox^ had the BPA incorporated at position 68 in the proximal Ub moiety, which also carried an N-terminal FAM label and a C-terminal His_6_-tag to prevent further conjugation; Ub_2_^Dist^ contained BPA at position 73 in the distal Ub moiety, together with a K48R mutation to prevent ubiquitin additions beyond the dimer (Figs. 1*B* and S1*A*). Ub_2_^Prox^ was synthesized by conjugating Ub(K48R) to FAM-Ub(H68BPA)-His_6_, while Ub_2_^Dist^ was synthesized by conjugating Ub(L73BPA, K48R) to FAM-Ub-His_6_, both using Uba1 and the K48-specific E2 enzyme Cdc34. Since the BPA-incorporation sites in Ub(L8BPA), Ub(H68BPA), and Ub(L73BPA) are close to, but not overlapping with the hydrophobic binding interface of ubiquitin, all three variants were expected to interact with Rpn1, Rpn10, and Rpn13 at the known ubiquitin-binding sites to allow for efficient crosslinking (Fig. 1, *C–E*).

Using a crosslinking assay with gel-based fluorescence readout, where a receptor is mixed with Ub(BPA), exposed to UV light (365 nm), and analyzed by SDS-PAGE, we tested how efficiently each mono-Ub probe as well as Ub_2_^Prox^ crosslinked to the three proteasomal receptors by tracking the FAM label on ubiquitin (Figs. 1, *F–H* and S1, *B–D*). Ub(H68BPA) was the most efficient mono-Ub probe, followed by Ub_2_^Prox^ for Rpn10, which is known to more tightly interact with diubiquitin *versus* monoubiquitin. In contrast, crosslinking to Rpn13 and Rpn1 was greatly reduced for Ub_2_^Prox^, likely because the Pru domain on Rpn13 and the T1 site on Rpn1 can accommodate only a single ubiquitin moiety at a time (6, 14), and the second, noncrosslinkable ubiquitin moiety in Ub_2_^Prox^ therefore acted as a competitor. As expected, Ub(I36BPA) with its BPA located more distant from the I44 patch showed weaker crosslinking than the other BPA-containing monomers. This reduced, yet still significant crosslinking was probably due to nonspecific interactions at the high protein concentrations used in this assay or due to conformational flexibility that for instance could bring the BPA36 of a ubiquitin bound to Rpn10’s UIM into close proximity to the UIM linker or the VWA domain. In summary, these initial data showed that the Ub(BPA) probes readily crosslink to known ubiquitin-binding subunits of the proteasome and can be used to capture these interactions *in vitro*.

### Ub(BPA) probes crosslink to receptors in a concentration-dependent manner

**Figure 2:**
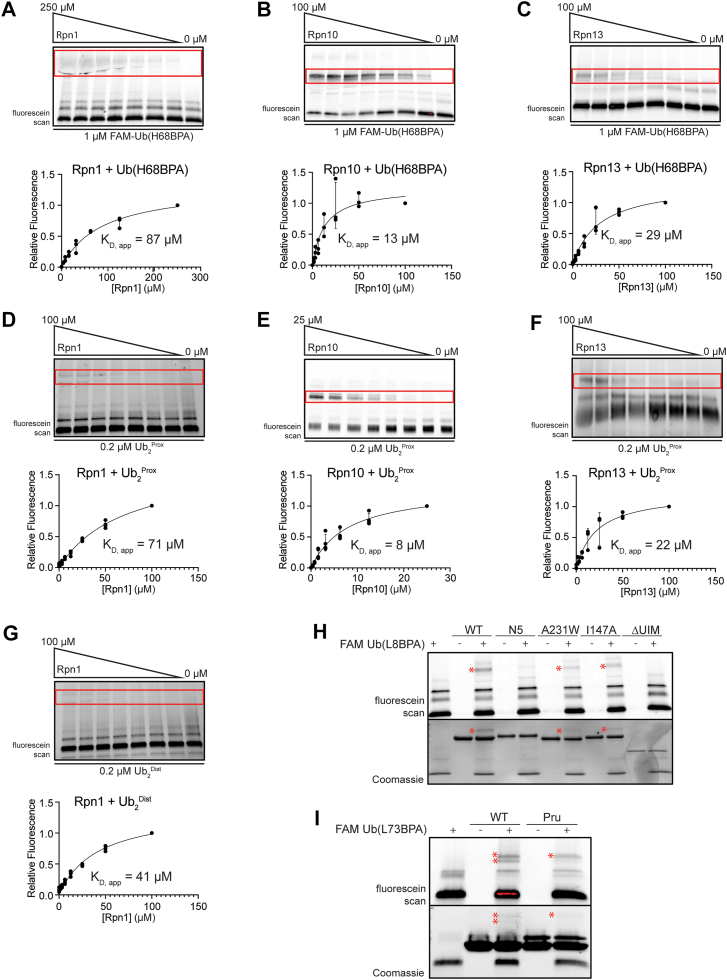
**Ub(BPA) probes crosslink at the expected affinities.***A*, *top*: representative gel of a titration of Rpn1 crosslinked to FAM-Ub(H68BPA). Crosslink band is boxed in *red*. *Bottom*: relative band intensities plotted against Rpn1 concentration and fit to a binding curve to determine K_D, app_. n = 3 technical replicates are shown. *B*, *top*: representative gel of a titration of Rpn10 crosslinked to FAM-Ub(H68BPA) as in (A). *Bottom*: binding curve and calculated K_D, app_ as in (A). n = 3 technical replicates are shown. *C*, *top*: representative gel of a titration of Rpn13 crosslinked to FAM-Ub(H68BPA) as in (A). *Bottom*: binding curve and calculated K_D, app_ as in (A). n = 3 technical replicates are shown. *D*, *top*: representative gel of a titration of Rpn1 crosslinked to Ub_2_^Prox^ as in (A). *Bottom*: binding curve and calculated K_D, app_ as in (A). n = 3 technical replicates are shown. *E*, *top*: representative gel of a titration of Rpn10 crosslinked to Ub_2_^Prox^ as in (A). *Bottom*: binding curve and calculated K_D, app_ as in (A). n = 4 technical replicates are shown. *F*, *top*: representative gel of a titration of Rpn13 crosslinked to Ub_2_^Prox^ as in (A). *Bottom*: binding curve and calculated K_D, app_ as in (A). n = 3 technical replicates are shown. *G*, *top*: representative gel of a titration of Rpn1 crosslinked to Ub_2_^Dist^ as in (A). *Bottom*: binding curve and calculated K_D, app_ as in (A). n = 3 technical replicates are shown. *H*, Crosslinking of FAM-Ub(L8BPA) to WT Rpn10 and its N5, A231W, I147A, and ΔUIM mutants. Crosslink bands are indicated with ∗. *I*, crosslinking of FAM-Ub(L8BPA) to WT Rpn13 and its Pru-domain mutant. Crosslink bands are indicated with ∗. BPA, p-benzoyl-L-phenylalanine; FAM, fluorescein amidite; Ub2, ubiquitin dimers; UIM, ubiquitin-interacting motif; Pru, pleckstrin-like receptor for ubiquitin.

To assess whether the Ub(BPA) probes could be used to measure apparent binding affinities, we titrated the receptor subunits against a constant Ub(H68BPA) concentration in our gel-based crosslinking assay and quantified the crosslinked band to obtain a saturation curve and apparent K_D_ (K_D, app_) (Fig. 2, *A*–*C*). The calculated K_D, app_ values are remarkably similar to those previously determined by NMR, ITC, or SPR (Table 1) (6, 19, 29, 30). Due to the rather inefficient reaction of BPA, crosslinking to the receptor is slow compared to the rates for ubiquitin binding and dissociation, and the extent of crosslinking is therefore directly modulated by the K_D_.

**Table 1:** Calculated K_D, app_ values compared to previously established K_D_ values

Receptor-ubiquitin probe	Apparent K_D_ (K_D, app_)	Published K_D_
Rpn10–Ub(H68BPA)	13 ± 7 μM	45 μM
Rpn10–Ub_2_^Prox^	8 ± 6 μM	13 μM
Rpn13–Ub(H68BPA)	29 ± 8 μM	91 μM
Rpn13–Ub_2_^Prox^	22 ± 8 μM	29 μM
Rpn1–Ub(H68BPA)	87 ± 20 μM	120 μM
Rpn1–Ub_2_^Prox^	71 ± 13 μM	12 μM
Rpn1–Ub_2_^Dist^	41 ± 10 μM	12 μM

Although the K_D, app_ for monomeric Ub(H68BPA) crosslinking to Rpn1 was less than 1.5-fold below the published K_D_, the K_D, app_ for Rpn10 and Rpn13 showed slightly larger deviations, ∼ 3 to 3.5-fold lower than previously reported values, which is likely due to the irreversible nature of the crosslink. Although there was no significant difference between the K_D, app_ values for monomeric Ub(H68BPA) and Ub_2_Prox interacting with Rpn10, Rpn13, or Rpn1, we observed a clear positional difference for the Ub_2_ in crosslinking to Rpn1, where Ub_2_^Dist^ had an almost 2-fold lower K_D, app_ than Ub_2_^Prox^ (Figs. 2, *D* and *G* and S2, *H–J*; Table 1). This agrees with the structure of K48-linked Ub_2_ bound to the T1 site of Rpn1 (Fig. 1*E*), showing that it is the distal moiety that primarily contacts the canonical ubiquitin-binding residues of the receptor.

Overall, our measured affinities indicate that fluorescence-based photo-crosslinking of the Ub(BPA) probes can serve as a valuable orthogonal method for the experimental determination of approximate binding affinities.

### Ub(BPA) crosslinking relies on canonical Ub-receptor interactions

To further assess whether Ub(BPA) crosslinking is dependent on specific ubiquitin−UBD interactions, we mutated residues in Rpn10 and Rpn13 that were previously shown to be responsible for binding (6, 7, 13, 14). Ubiquitin interactions with Rpn10 had been mapped to five amino acids in the UIM (30), and our mutation of all five residues (L228N, A229N, M230N, A231N, and L232N, labeled “Rpn10 N5”) indeed completely abolished observable crosslinking (Fig. 2*H*). Mutation of just one of these residues, A231W, led to a considerable decrease in the crosslinking intensity. In contrast, mutation of I147, which is located in the VWA domain of Rpn10 and was previously proposed as part of additional ubiquitin-binding interface (31), had no major effect on crosslinking, suggesting that the VWA domain is not involved in ubiquitin binding for substrate recruitment to the proteasome. Consistently, Ub(BPA) showed no detectable crosslinking to the isolated VWA domain (ΔUIM) (Fig. 2*H*).

For Rpn13, we mutated five Pru-domain residues involved in ubiquitin binding (E41K, E42K, L43A, F45A, and S93D) and observed a significant reduction in crosslinking compared to the WT Rpn13 (Fig. 2*I*). In addition to the mutation-sensitive crosslink band, Rpn13 exhibited a secondary band at higher molecular weight that we attribute to nonspecific crosslinking, either due to the high protein concentrations used in our assay or the exposed interfaces on isolated Rpn13 that are otherwise used for receptor interactions with the proteasome.

### LC-MS/mass spectrometry (MS) can be used to identify ubiquitin-crosslinked peptides in Rpn10

**Figure 3:**
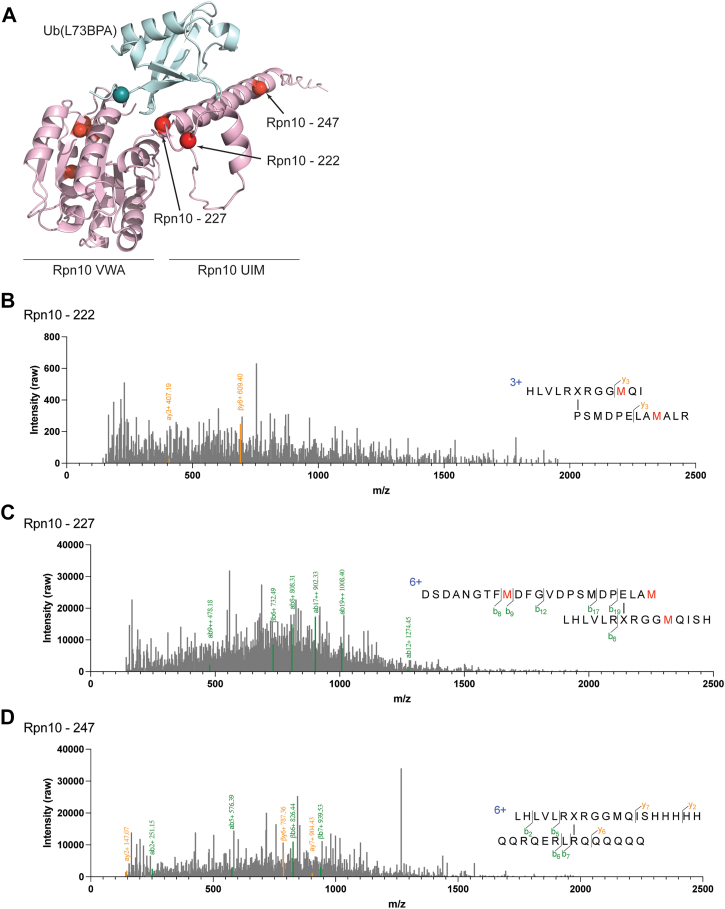
**Identification of crosslinked peptides from Rpn10 using LC-MS/MS.***A*, AlphaFold structural model of Rpn10 (*pink*) bound to Ub (*light blue*). L73BPA in ubiquitin is shown as a *teal sphere* and identified crosslinks in Rpn10 are shown as *red spheres*. *B*, *left*: spectrum of an identified peptide containing a crosslink at Rpn10 222. *Right*: schematic of the identified peptide. BPA is indicated as ‘X’. *C*, *left*: spectrum of an identified peptide containing a crosslink at Rpn10 227. *Right*: schematic of identified peptide. BPA is indicated as ‘X’. *D*, *left*: spectrum of an identified peptide containing a crosslink at Rpn10 247. *Right*: schematic of identified peptide. BPA is indicated as ‘X’. MS, mass spectrometry; BPA, p-benzoyl-L-phenylalanine.

Rpn10 is the best-characterized ubiquitin receptor and thus can serve as a model system for testing whether Ub(BPA) crosslinking can be combined with MS to identify specific binding sites on the proteasome. We therefore crosslinked Ub(L73BPA) to Rpn10, digested the reaction products with chymotrypsin, ran LC-MS/MS analyses, and used the pLink software (32) to assign specific spectra to crosslinked peptides. This analysis identified seven Rpn10 peptides, whose mapping on the AlphaFold model of the ubiquitin-bound Rpn10 structure revealed that three of them lie in or adjacent to the UIM (P222, E227, and R247), while the other four peptides stem from crosslinks to the VWA domain (A5, V49, A94, and K104; Fig. 3*A*). The crosslinks in and near the UIM confirm the UIM as the ubiquitin-binding site (Figs. 3, *B–D* and S3). That the identified residues do not agree with those previously shown to directly contact ubiquitin is expected, given our experimental design, with BPA positions on ubiquitin bordering, yet not overlapping with the interaction interface. Ub(BPA) crosslinking can thus detect broad regions of interaction, but not pinpoint specific residues. The observed crosslinks to the VWA domain are likely nonspecific and caused by Rpn10 being outside the proteasome context, as these positions are buried when Rpn10 is bound to the 19S RP. Due to its high sensitivity, MS detected those off-target crosslinks, whereas no significant crosslinking was observed for the Rpn10 ΔUIM construct in our gel-based assay (Fig. 2*H*).

### Ub(BPA) probes identify novel Ub receptors on the 26S proteasome

**Figure 4:**
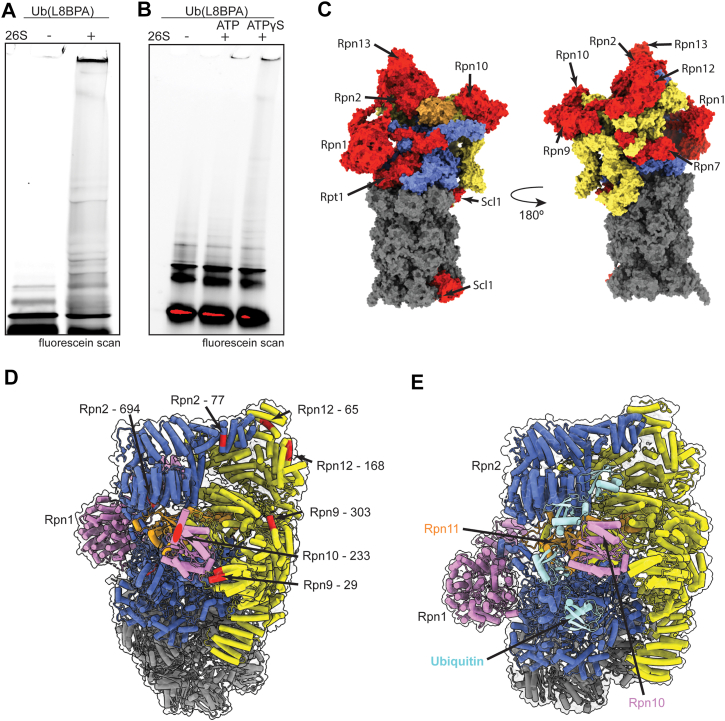
**Ub(BPA) probes identify novel ubiquitin receptors on the 26S proteasome.***A*, gel of Ub(L8BPA) crosslinked to purified 26S proteasome imaged with fluorescein-fluorescence scan. *B*, crosslinking of Ub(L73BPA) to purified 26S proteasome in the presence of ATP or in the presence of ATPγS imaged by fluorescein-fluorescence scan. *C*, front view (*left*) and back view (*right*) of the 26S proteasome structure (PDB: 6FVT) with crosslinked subunits shown in *red*, Rpn11 in *orange*, noncrosslinked base subunits in *blue*, noncrosslinked lid subunits in *yellow*, and noncrosslinked core subunits in *gray*. *D*, view from the *top right* of the front orientation shown in (C). All subunits are shown in *tube* representation inside a transparent proteasome surface and colored as in (C), with identified crosslinked peptides colored in *red*. Rpn10’s UIM (unresolved in this structure, PDB: 9CGC) is modeled also as a *pink cylinder*. Crosslinks surrounding the groove atop the 19S RP are labeled. E, structure of the human 26S proteasome with a bound K11/K48-linked branched ubiquitin chain (PDB: 8JTI). Ubiquitin moieties are shown in *cyan*, Rpn1 and Rpn10 in *pink*, other base subunits in *blue*, Rpn11 in *orange*, other lid subunits in *yellow*, and core subunits in *gray*. BPA, p-benzoyl-L-phenylalanine; PDB, Protein Data Bank; UIM, ubiquitin-interacting motif; RP, regulatory particle.

To reveal novel ubiquitin receptors and binding sites on the 26S proteasome, we crosslinked Ub(L73BPA) to endogenous proteasome holoenzymes from yeast. These proteasomes were purified by affinity chromatography, using a FLAG-tag on Rpn11, and size-exclusion chromatography (SEC) (Fig. S4, *A* and *B*), such that all potentially associated proteins, including the shuttle factors Dsk2 and Rad23, the deubiquitinase Ubp6, or the E3 ligase Hul5, were maintained. Exposing these proteasomes in ATP to UV-induced crosslinking with FAM-Ub(L73BPA) for 30 min produced a continuous smear on the SDS-PAGE gel up to higher molecular weights, indicating that the probe reacted with multiple components of the proteasome (Fig. 4*A*). We also found that the proteasome crosslinking pattern on the gel changed dramatically in the presence of nonhydrolyzable ATPγS (Fig. 4*B*), which is known to shift the conformational equilibrium from the resting (s1) state to a processing (s4) state (33, 34). These changes suggest a difference in the availability of Ub-binding sites between resting and processing states, and support a model where the conformational transitions to non-s1 states after substrate insertion into the ATPase motor facilitate ubiquitin release from certain receptors or interfaces (35). To identify which subunits were crosslinked to our ubiquitin probe, we developed a workflow for sample preparation that capitalized on the His_6_-tag present at the C terminus of the probe (Fig. S4*C*). Purified proteasomes were crosslinked to Ub(L73BPA) under the standard conditions used in our other assays and then completely denatured with 6 M guanidinium chloride. The Ub probe and any crosslinked proteins were enriched by Ni-NTA affinity purification, trypsinized, and analyzed by LC-MS/MS. The pLink search for crosslinked peptides varied between individual attempt, and each proteasomal subunit was therefore searched four times. When attempting to replicate pLink search results, we found that hits were not always consistent between pLink searches, thus for this analysis, any particular peptide was only considered a positive hit if it was present in at least three of the four independent searches.

Using this workflow, we identified crosslinked peptides from numerous subunits of the 19S RP, including the canonical Ub receptors Rpn1, Rpn10, and Rpn13, the base subunits Rpn2 and Rpt1, the lid subunits Rpn7, Rpn9, and Rpn12, and the α1 subunit of the 20S core (Fig. 4*C*; Table 2; Fig. S7). Rpn10 had a crosslink at A233, in the middle of its UIM (Fig. S5*A*), while Rpn13 showed a crosslink at E60, located adjacent to the Pru residues (Fig. S5*B*). The crosslinked residue in Rpn1, L873, lies in a cleft between Rpn1’s toroid and N-terminal domains, near but not immediately adjacent to the T1 site (Fig. S5*C*). Given this position of L873, we assume that other, more exposed residues may have been responsible for the robust Ub crosslinking observed for Rpn1 in our gel-based assays, yet were not identified due to the intricacy of detecting crosslinked peptides by MS. In addition, we identified peptides from Ubp6 and Hul5 (Table 2). The crosslink on Ubp6 is located adjacent to its ubiquitin-binding site (Fig. S6*A*), whereas the crosslink on Hul5 lies in a secondary site predicted by AlphaFold2 to interact with the E2 enzyme Ubc4 (Fig. S6*B*).

**Table 2:** Crosslinks identified from pLink searches

Subunit	Crosslinked residue(s)
Rpn1	873
Rpn2	77, 694
Rpn7	23
Rpn9	29, 303
Rpn10	233
Rpn12	65, 168
Rpn13	60
Rpt1	148
Scl1	111
Ubp6	330
Hul5	697, 698

There were no crosslinks to any other proteasomal subunits observed.

Mapping these crosslinks onto the structure of the 26S proteasome in the resting s1 state revealed that several contact points surround a groove formed by Rpn2, Rpn9, Rpn10, and Rpn12 on top of the 19S RP (Fig. 4*D*). This groove may serve as a binding region for extended or branched ubiquitin chains that interact with Rpn10’s UIM, which lies just below the groove. Indeed, recent cryo-electron microscopy structures of the human 26S proteasome with branched K11/K48-linked chains show ubiquitin moieties positioned along the same groove (36, 37) (Fig. 4*E*), in nice agreement with our crosslinking data. Additional crosslinks we observed in the back of this groove suggest that ubiquitin chains longer than the ones used in these structural studies may extend further toward the back of the proteasome. Together with the structural data, our crosslinking results suggest that the groove flanked by Rpn2, Rpn9, Rpn10, and Rpn12 serves as a novel ubiquitin-binding region on top of the 26S proteasome. Moreover, these findings further support the potential of Ub(BPA) crosslinking for mapping ubiquitin-interacting sites on macromolecular complexes.

## Discussion

Due to ubiquitin’s promiscuity and often low binding affinities, its interactions with other proteins have been difficult to capture and characterize biochemically. The use of traditional chemical crosslinkers to capture nonenzymatic ubiquitin interactions is limited by large crosslinking radii and relies on the presence of specific functional groups to crosslink between. Our results show that BPA incorporation near the canonical interface of ubiquitin allows the development of specific interaction probes. Ub(BPA) crosslinking can thereby be used not only to identify novel binding sites, but also to estimate binding affinities with a gel-based fluorescence assay that provides K_D_ values similar to those from conventional methods, like NMR, ITC, or SPR, which require specialized equipment and expertise.

Our assay is not an equilibrium method due to the irreversibility of crosslinking. However, BPA has relatively low reactivity and can be repeatedly activated over prolonged periods of time (22), such that the extent of crosslinking is modulated by the preequilibrium of ubiquitin binding and dissociation. The derived K_D, app_ values are therefore in reasonable agreement with the binding affinities determined by true equilibrium measurements. Furthermore, our data show that positional incorporation of Ub(BPA) into a dimer revealed different K_D, app_ values, providing a platform for determining the binding contributions of particular ubiquitin moieties within a chain.

BPA is more specific than traditional chemical crosslinkers due to its short crosslinking radius, yet retains promiscuity by reacting indiscriminately with C−H bonds. BPA crosslinking is therefore an effective approach for capturing low-to medium-affinity interactions, such as ubiquitin binding. When we applied Ub(BPA) crosslinking to the 26S proteasome, we detected all three Ub receptors, indicating that these BPA probes nicely complement conventional chemical crosslinkers (38) and alternative crosslinkable ubiquitin variants (39). We did, however, observe the challenges of using MS to identify crosslinks (40), which required multiple searches to confirm that a peptide was indeed present in a dataset. We also identified crosslinks to several other proteasome-resident proteins that are known to interact with ubiquitin, such as Ubp6 and Hul5, highlighting how Ub(BPA) can capture small populations of canonical ubiquitin-protein interactions *in vitro*. Moreover, our crosslinked peptide search identified additional binding sites, suggesting that a groove on the top of the RP interacts with ubiquitin. Our analysis was aided by two recent structures showing that K11/K48-branched Ub chains bind to the human 26S proteasome through the front of this groove (36, 37). These structures thus provide some validation of our photo-crosslinking MS hits and indicate that this is a conserved ubiquitin-binding groove shared between human and yeast proteasomes. It is possible that this groove accommodates chains with specific linkages or topologies to select certain substrates for degradation.

Future work will have to investigate the context of ubiquitin binding to the groove on top of the 26S proteasome. In addition, our Ub(BPA) probes can be used as a starting point to synthesize more complex probes with Ub(BPA) incorporated into Ub chains that contain certain physiologically relevant features, including branches. These more complex probes and the Ub(BPA) presented here could be applied to other systems for mapping ubiquitin-binding sites and determining which moieties in chains are contacted. However, it is important to note that several of our identified crosslinks, especially for insolated receptor subunits, are unlikely to be physiologically relevant, given their expected steric clashes with other subunits in the context of the 26S proteasome holoenzyme. This highlights how photo-crosslinking MS can capture off-target false positives and that it is necessary to validate identified crosslinks through additional mutational, biochemical, or structural studies when applying these probes to map novel ubiquitin interactions.

## Experimental procedures

### Expression and purification of Ub(BPA)

BL21 DE3∗ competent cells were cotransformed with vectors coding for the BPA aminoacyl-tRNA synthetase (MjTyrRS)/tRNA pair and ubiquitin containing an amber stop codon in the position desired for BPA incorporation, Ub(BPA) (21, 28). Cells were grown in dYT media at 37 °C until OD_600_ ∼0.6, then pBPA dissolved in NaOH was added to a final concentration of 2 mM, and cells were induced with 1 mM final IPTG overnight at 18 °C. Cells were harvested by centrifugation and resuspended in NiA buffer (50 mM hepes pH 7.6, 250 mM NaCl, and 20 mM imidazole) with protease inhibitors (AEBSF, leupeptin, pepstatin, and aprotinin). Pellets were lysed by sonication, and the lysate was clarified by centrifugation. His-tagged Ub(BPA) proteins were affinity purified using Ni-NTA resin, washed with NiA buffer and eluted with NiB buffer (50 mM hepes pH 7.6, 250 mM NaCl, and 250 mM imidazole), followed by SEC using a Superdex 75 16/600 column (Cytiva) equilibrated in gel-filtration (GF) buffer (50 mM hepes pH 7.6, 50 mM NaCl, 80 mM KCl, and 10 mM MgCl_2_, 5% glycerol). Fractions were concentrated using a 3K cutoff concentrator. Ub(BPA) variants without a His_6_ tag were purified by acetic acid precipitation, followed by overnight dialysis in buffer A1 (50 mM NaOAc), ion-exchange chromatography using a 5 ml HiTrap SP FF with buffer A1 and 0 to 70% buffer B1 (50 mM NaOAc, 500 mM NaCl) over 20 column volumes, and SEC using a Superdex 75 16/600 column (Cytiva) equilibrated in GF buffer. Fractions were concentrated using a 3K cutoff concentrator.

### Fluorescent labeling of Ub(BPA)

Purified Ub(BPA) was mixed with 20 μM sortase, 500 μM fluorescein-LPETGG, and 10 mM CaCl_2_, and incubated at room temperature for 1 h. The sample was then purified by Ni-NTA affinity using a 1 ml HisTrap HP column (Cytiva) equilibrated with NiA buffer, and eluted using NiB buffer. It was further purified by SEC using a Superdex S75 Increase column (Cytiva) equilibrated in GF buffer.

### Synthesis of K48-linked di-Ub chains

K48-linked Ub_2_ probes were synthesized by incubating 500 μM Ub(K48 R) and 10 μM FAM-Ub(BPA)-His_6_ (for Ub_2_^Prox^) or 500 μM Ub(BPA, K48 R) and 10 μM FAM-Ub-His_6_ (for Ub_2_^Dist^) with 0.3 μM mouse E1, 3 μM Cdc34, and 1 mM ATP in a total volume of 200 μl at 37 °C overnight (41). The Ub_2_ probes were cleaned up by Ni-NTA affinity purification using a 1 ml HisTrap HP column (Cytiva), followed by SEC using a Superdex 75 Increase 10/300 (Cytiva) equilibrated in GF buffer. Fractions containing Ub_2_ probe were collected and concentrated using a 3K cutoff concentrator.

### Expression and purification of proteasome subunits

BL21 DE3∗ competent cells were transformed with vectors containing His-tagged Rpn1, Rpn10, or Rpn13. Cells were grown in dYT media to OD_600_ ∼0.6 at 37 °C, then induced with 1 mM IPTG, and incubated overnight at 18 °C. Cells were harvested by centrifugation and resuspended in NiA buffer (50 mM hepes pH 7.6, 250 mM NaCl, and 20 mM imidazole) with protease inhibitors (AEBSF, leupeptin, pepstatin, and aprotinin). Pellets were lysed by sonication and lysate was clarified by centrifugation. His-tagged proteasome subunits were purified by affinity purification using Ni-NTA resin washed with NiA buffer and eluted with NiB buffer (50 mM hepes pH 7.6, 250 mM NaCl, and 250 mM imidazole), followed by SEC using a Superdex 75 16/600 column (Rpn10, Rpn13) or a Superdex 200 16/600 (Rpn1) equilibrated in GF buffer (50 mM hepes pH 7.6, 50 mM NaCl, 80 mM KCl, 10 mM MgCl_2_, and 5% glycerol). Fractions were concentrated and concentration was determined by absorbance A_280_ measured on a Nanodrop.

### Expression and purification of yeast proteasome

Yeast 26S proteasome was purified from the YYS40 strain containing FLAG-Rpn11.^42^ Yeast cells were streaked from a glycerol stock on a yeast peptone dextrose medium (YPD) plate and incubated at 30 °C until colonies appeared. Starter cultures of 5 ml were inoculated in YPD using one colony per starter and incubated at 30 °C overnight. Starter cultures of 50 ml with YPD media were inoculated with the 5 ml starter cultures and incubated at 30 °C overnight. YPD media of 1 l was inoculated with 25 ml starter culture and incubated at 30 °C for 3 days. Cells were collected by centrifugation, resuspended in buffer, and popcorned into liquid nitrogen. After cryogrinding of the cells, the lysate powder was thawed in lysis buffer (60 mM hepes pH 7.6, 25 mM NaCl, 25 mM KCl, 5 mM MgCl_2_, 0.5 mM EDTA, 5% glycerol, supplemented with 0.01% NP-40, 0.05 mg/ml creatine phosphokinase, 16 mM creatine phosphate, and 2 mM ATP). Lysate was clarified at 15,000 rpm for 30 min, the supernatant was incubated with FLAG resin equilibrated with yeast GF buffer (60 mM hepes pH 7.6, 25 mM NaCl, 25 mM KCl, 5 mM MgCl_2_, 0.5 mM EDTA, 5% glycerol, and 0.5 mM ATP) at 4 °C for 30 min. The resin was washed with yeast GF four times, the proteasome was eluted using yeast GF buffer supplemented with 0.15 mg/ml FLAG peptide, and the solution was concentrated in a 10K cutoff concentrator to a volume < 500 μl. Proteasome was cleaned up by SEC using a Superose 6 Increase 10/300 column (Cytiva) equilibrated with FPLC buffer (25 mM hepes pH 7.6, 25 mM NaCl, 25 mM KCl, 5 mM MgCl_2_, 0.5 mM EDTA, 5% glycerol, 0.5 mM ATP, and 1 mM DTT) and concentrated to 100 μl using a 10K cutoff concentrator. Concentration was determined by Bradford.

### Gel-based crosslinking assay

Purified Ub(BPA) probe and proteasome subunits or 26S proteasome holoenzyme were diluted to indicated concentrations in GF buffer in a total volume of 10 μl. Reactions were exposed to UV light (365 nm) on ice for 30 min. Crosslinking was analyzed by SDS-PAGE and imaging of the gels by fluorescein fluorescence, before staining with Coomassie. Band intensities on gels were quantified based on the intensity of crosslinked relative to the sum of crosslinked and uncrosslinked species using BioRad Image Lab, and data were analyzed with Prism 10 (GraphPad Software).

### Rpn10 crosslinking

An 80 μl solution of 3.25 μM Rpn10 was crosslinked to 2.5 μM Ub(L73BPA) by incubating on ice under UV light (365 nm) for 30 min. The sample was buffer-exchanged into 50 mM ammonium bicarbonate pH 8.0 using a 3K cutoff concentrator and the volume was reduced to 50 μl.

Crosslinking and denaturing enrichment of the 26S proteasome1.5 μM yeast 26S proteasome was crosslinked to 1 μM Ub(L73BPA) in 40 μl by incubation on ice under UV light (365 nm) for 30 min. The sample was diluted to 1 ml in denaturing buffer (50 mM hepes pH 7.6, 20 mM imidazole, and 6 M guanidine hydrochloride) and incubated at room temperature for 15 min. The sample was loaded onto a 1 ml HiTrap column (Cytiva) equilibrated with denaturing buffer, washed twice with 5 ml denaturing buffer, and eluted with 7 ml of 50 mM hepes pH 7.6, 250 mM imidazole, and 6 M guanidine hydrochloride. The sample was concentrated in a 3K cutoff concentrator and buffer exchanged into 50 mM ammonium bicarbonate (pH 8.0), before reducing the volume to 50 μl.

### Tryptic digestion

The sample volume was brought to 60 μl with 50 mM ammonium bicarbonate (pH 8.0) and 25% acetonitrile. Tris(2-carboxyethyl)phosphine was added to a final concentration of 5 mM, and the sample was incubated at room temperature for 20 min. Iodoacetamide was added to a final concentration of 10 mM, and the sample was incubated at room temperature in the dark for 20 min. CaCl_2_ was added to a final concentration of 1 mM, and the sample was incubated overnight at 37 °C with at least 1 μg trypsin. The sample was acidified with formic acid to 5%, diluted, and reduced in volume to 10 μl in a Speedvac. Rpn10 was digested with chymotrypsin following a similar protocol.

### High pH offline fractionation

In-gel trypsin-digested peptides from crosslinked 26S proteasome samples were filtered with a 0.22 μm centrifugal filter (Millipore UFC30GVNB) and manually loaded on an in-house prepared fused silica capillary column (250 μm × 35 cm) with Kasil outlet end. The column was packed with ReproSil pHoenix C18-3.0 μm high PH resin (Dr Maisch GmbH). Peptides were eluted at a flow rate of 2 μl/min using a linear gradient of 2 to 40% buffer B in 140 min (buffer A: 0.1% triethylamine pH 10.0, 5% acetonitrile in water; buffer B: 0.1% triethylamine pH 10.0, 95% acetonitrile in water) with an Agilent 1200 nanoflow LC. Samples were collected every 3 min during the gradient using a fraction collector (Biorad 2110). The 60 fractions were concatenated to eight fractions and acidified with formic acid to pH 2.0, before reducing their volume to 10 μl in a Speedvac.

### LC-MS/MS

Trypsin-digested peptides were analyzed by online capillary nanoLC-MS/MS using a 25 cm reversed phase column and a 10 cm precolumn fabricated in-house (75 μm inner diameter, packed with ReproSil-Gold C18–1.9 μm resin (Dr Maisch GmbH)) that was equipped with a laser-pulled nano-electrospray emitter tip. The precolumn used 3.0 μm packing (Dr Maisch GmbH). Peptides were eluted at a flow rate of 300 nl/min using a linear gradient of 2 to 40% buffer B in 140 min (buffer A: 0.05% formic acid, 5% acetonitrile in water; buffer B: 0.05% formic acid, and 95% acetonitrile in water) in an Easy-nLC1200 nanoLC system (Thermo Fisher Scientific). Peptides were ionized using a FLEX ion source (Thermo Fisher Scientific) and electrospray ionization into an Fusion Lumos Tribrid Orbitrap Mass Spectrometer (Thermo Fisher Scientific). Data were acquired in Orbi-trap mode. Instrument method parameters were as follows: MS1 resolution, 120,000 at 200 m/z; scan range, 350 to 1600 m/z; MS2 resolution/scan rate, ∼ 20 to 30 ms; MS1 AGC target, ∼ 2 e^5^ – 4 e^5^; MS2 AGC target, 5 e^5^; MS1 maximum injection time, 50 ms; MS2 maximum injection time, 35 ms; charge state range, 2 to 7. The top 20 most-abundant ions were subjected to collision-induced dissociation with a normalized collision energy of 35%, activation q 0.25, and precursor isolation width 2 m/z. Dynamic exclusion was enabled with a repeat count of 1, a repeat duration of 30 s, and an exclusion duration of 20 s.

### Crosslinked peptide search

Crosslinked peptides were identified using the pLink software (32). MGF files exported from PEAKS (Bioinformatics Solutions Inc.) were searched with the following parameters: crosslinker set as BPA, enzyme type was set to nonspecific, up to three missed cleavages, peptide mass 600 to 10,000 Da, peptide length 4 to 100 residues, precursor tolerance set to 10 ppm, fragment tolerance set to 10 ppm, fixed carbamidomethyl C modification, variable oxidation M modification, separate FDR >5% at peptide pairs level, with a reference database containing the Ub(BPA) sequence and the sequence of 1 to 2 proteasome subunits. Our version of pLink crashed when searching large sequence libraries, leading to the use of small reference databases over many searches. Each subunit was searched 4 times, at least once as the only reference sequence other than Ub(BPA). Crosslinked peptides that were identified in 75 to 100% of searches for that subunit were considered true positive hits. Crosslinked peptides identified in 50% or fewer of searches were considered false positives. Proteasome subunits or associated proteins that did not have any identified crosslinks after two searches were not searched more times. If any two identified peptides were assigned to the same peaks, the one with the higher score assigned by pLink was considered real. Spectra corresponding to the crosslinked peptides were analyzed in pLabel and plotted in Prism 10 (GraphPad Software).

## Data availability

Further information and requests for resources and reagents should be directed to and will be fulfilled by the lead contact, Andreas Martin (a.martin@berkeley.edu). Data generated or analyzed during this study are included in this article and the Supporting Information. The raw MS data were deposited into the ProteomeXchange Consortium *via* the PRIDE partner repository under the dataset identifier PXD075114. This paper does not report original code. All constructs are available from the lead contact upon request and completion of a Material Transfer Agreement.

## Supporting information

This article contains supporting information.

## Conflict of interest

The authors declare that they have no conflicts of interest with the contents of this article.
