# Supplementary material for: Identification of novel ubiquitin receptors on the 26S proteasome by photo-crosslinking mass spectrometry
Source: J Biol Chem. 2026 Apr 22;302(6):111481. doi: 10.1016/j.jbc.2026.111481 (PMC13208731; doi:10.1016/j.jbc.2026.111481)
Supplement: Tables and Figures [file mmc1.pdf]

Supporting Information:

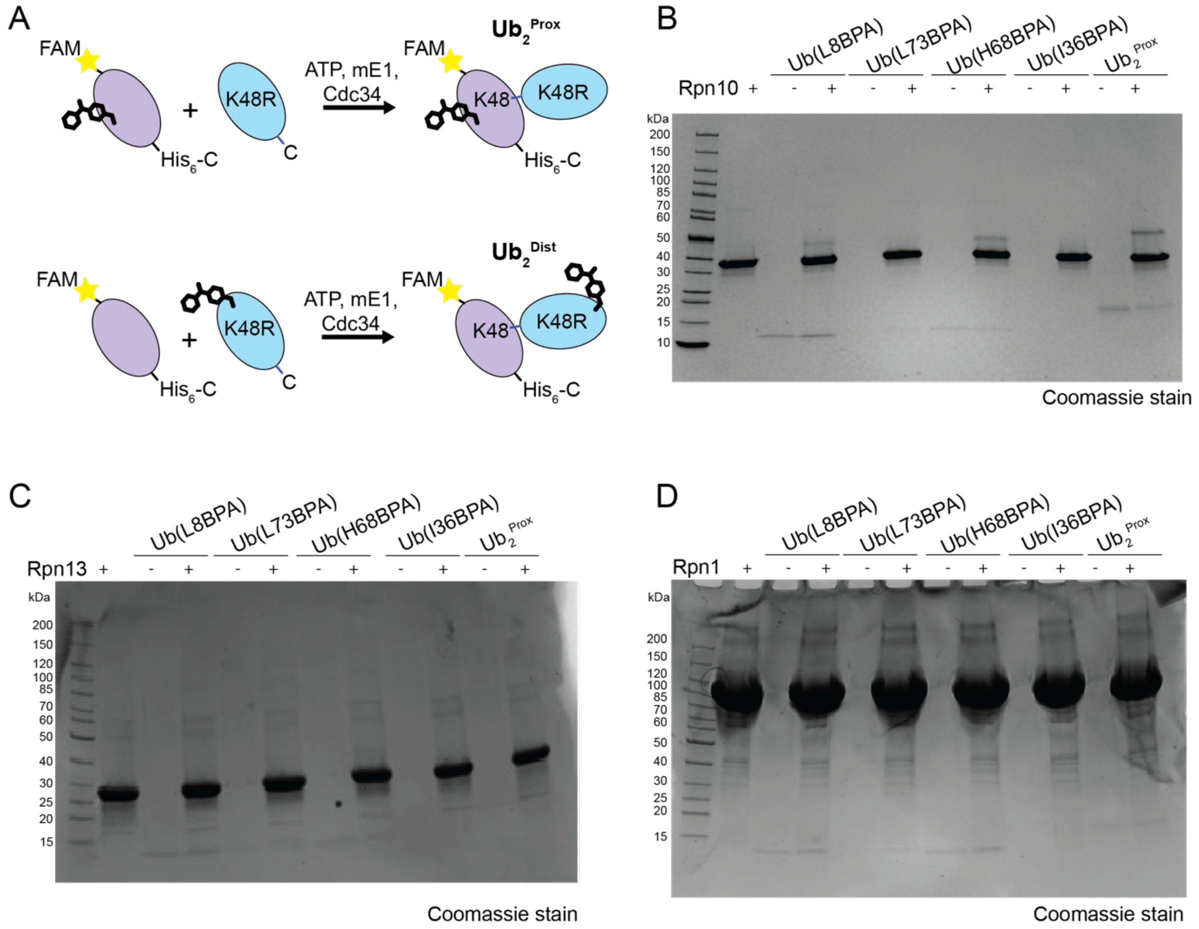

Supporting Figure S1: Generation and crosslinking of Ub(BPA) probes. A) Schematic of Ub_2^{Prox} and Ub_2^{Dist} probe production via Ub(BPA)-Ub conjugation, with Prox and Dist indicating the BPA position in the proximal or distal ubiquitin of the resulting K48-linked dimer. To direct the conjugation and prevent formation of larger oligomers, the proximal, FAM-labeled Ub carried a C-terminal His_6 tag, while the distal Ub contained a K48R mutation. **B)** Coomassie-stained SDS-PAGE gel of Ub(L8BPA), Ub(L73BPA), Ub(H68BPA), Ub(I36BPA), and Ub_2^{Prox} crosslinking to Rpn10, related to Figure 1F. **C)** Coomassie-stained SDS-PAGE gel of Ub(L8BPA), Ub(L73BPA), Ub(H68BPA), Ub(I36BPA), and Ub_2^{Prox} crosslinking to Rpn13, related to Figure 1G. **D)** Coomassie-stained SDS-PAGE gel of Ub(L8BPA), Ub(L73BPA), Ub(H68BPA), Ub(I36BPA), and Ub_2^{Prox} crosslinking to Rpn1, related to Figure 1H.

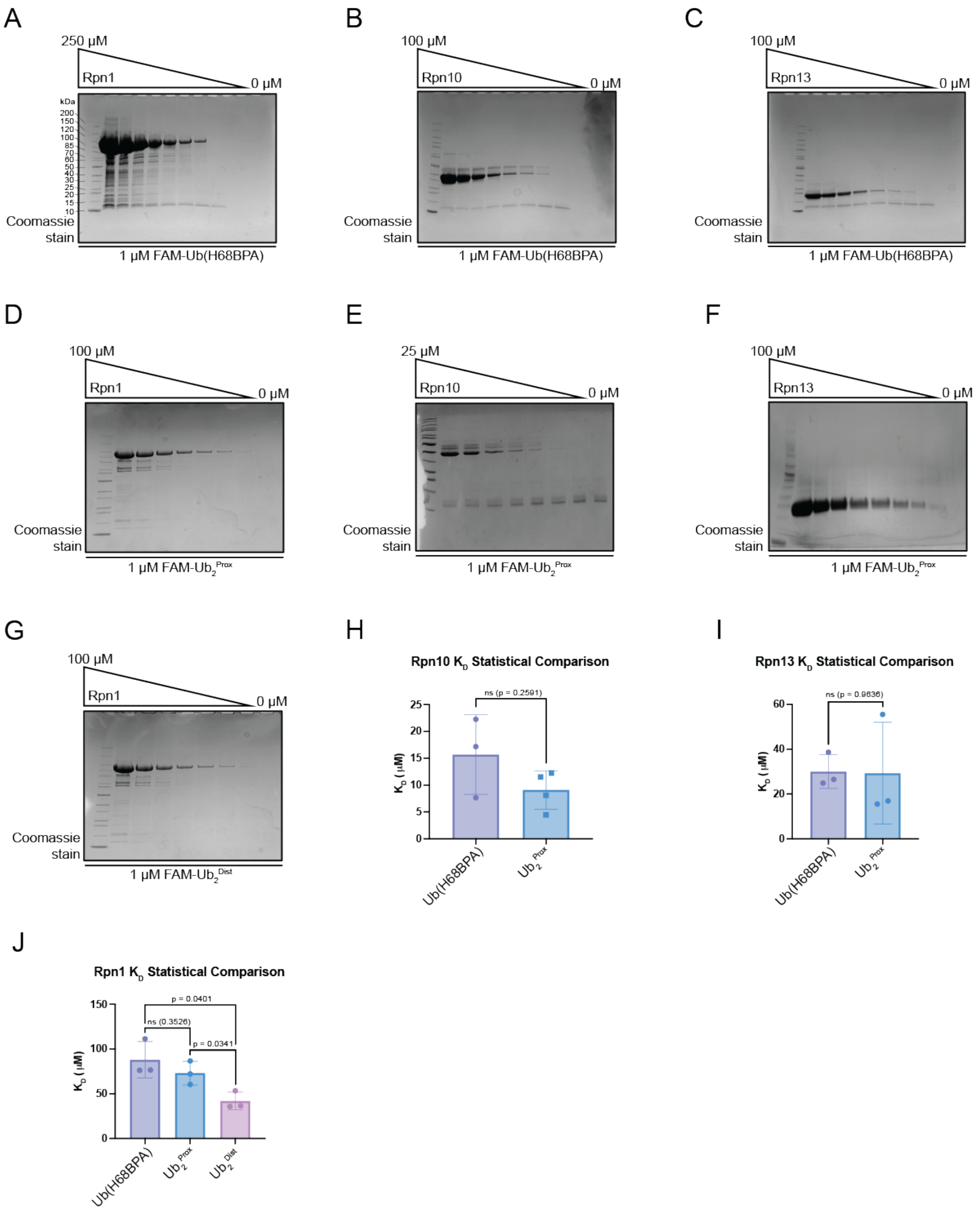

Supporting Figure S2: Coomassie-stained SDS-PAGE gels for the analyses of Ub(BPA) crosslinking to proteasomal receptors. **A)** Representative Coomassie-stained gel of Ub(H68BPA) crosslinked to various concentrations of Rpn1, related to Figure 2A. **B)** Representative Coomassie-stained gel of Ub(H68BPA) crosslinked to various concentrations of Rpn10, related to Figure 2B. **C)** Representative Coomassie-stained gel of Ub(H68BPA) crosslinked to various concentrations of Rpn13, related to Figure

2C. **D)** Representative Coomassie-stained gel of Ub_2^{Prox} crosslinked to various concentrations of Rpn1, related to Figure 2D. **E)** Representative Coomassie-stained gel of Ub(H68BPA) crosslinked to various concentrations of Rpn10, related to Figure 2E. **F)** Representative Coomassie-stained gel of Ub(H68BPA) crosslinked to various concentrations of Rpn13, related to Figure 2F. **G)** Representative Coomassie-stained gel of Ub_2^{Dist} crosslinked to various concentrations of Rpn1, related to Figure 2G. **H)** Unpaired T-test of K_Ds from crosslinking Rpn10 to Ub(H68BPA) and Ub_2^{prox}. P-value = ns. **I)** Unpaired T-test of K_Ds from crosslinking Rpn13 to Ub(H68BPA) and Ub_2^{prox}. P-value = ns. **J)** Unpaired T-test of K_Ds from crosslinking Rpn1 to Ub(H68BPA), Ub_2^{prox}, and Ub_2^{dist}. P-value comparing Ub_2^{prox} and Ub_2^{dist} = 0.0341.

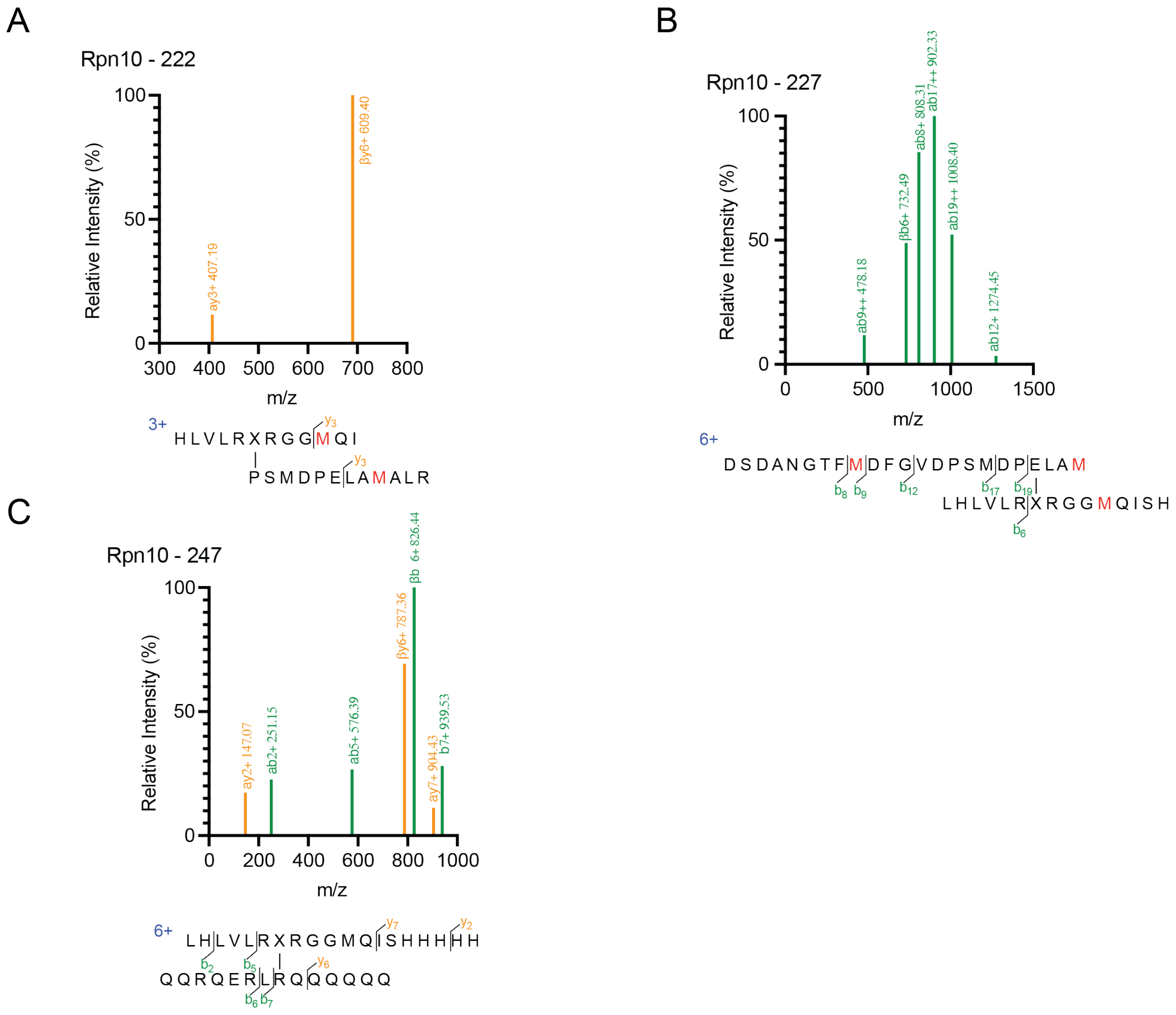

Supporting Figure S3: Identification of crosslinked peptides from Rpn10. A) Left: Spectrum of an identified peptide containing a crosslink at Rpn10 222 as in Figure 3B. Right: Schematic of the identified peptide. BPA is indicated as 'X'. **B)** Left: Spectrum of an identified peptide containing a crosslink at Rpn10 227 as in Figure 3C. Right: Schematic of identified peptide. BPA is indicated as 'X'. **C)** Left: Spectrum of an identified peptide containing a crosslink at Rpn10 247 as in Figure 3D. Right: Schematic of identified peptide. BPA is indicated as 'X'.

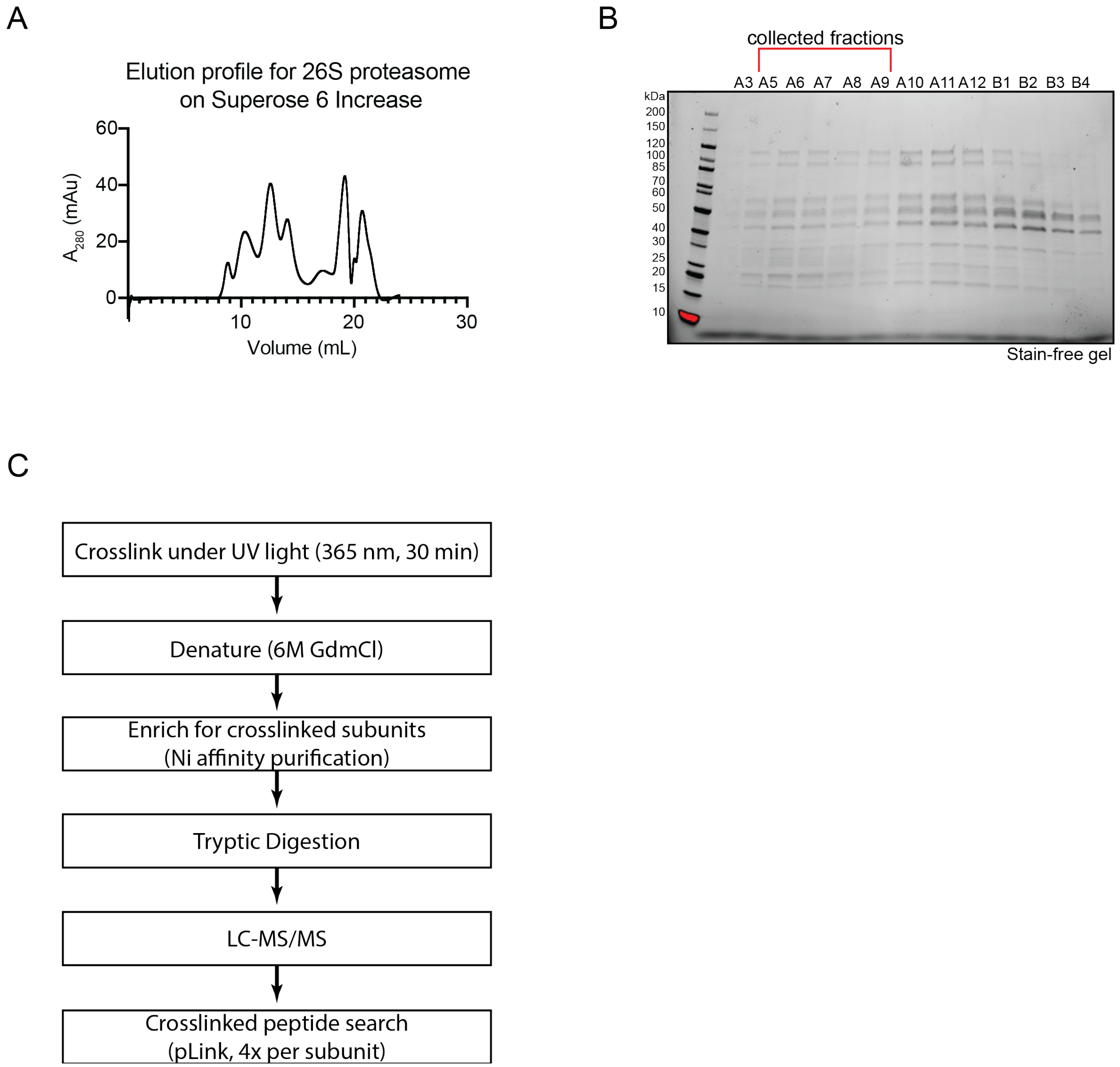

Supporting Figure S4: Purification and crosslinking of 26S proteasomes. A) Size-exclusion chromatogram of the yeast 26S proteasome eluting from a Superose 6 Increase column. **B)** Stain-free SDS-PAGE gel of fractions from size-exclusion chromatography purification of 26S proteasome shown in panel (A). Collected fractions are indicated above. **C)** Workflow for the photo-crosslinking mass spectrometry experiments and identification of crosslinked peptides.

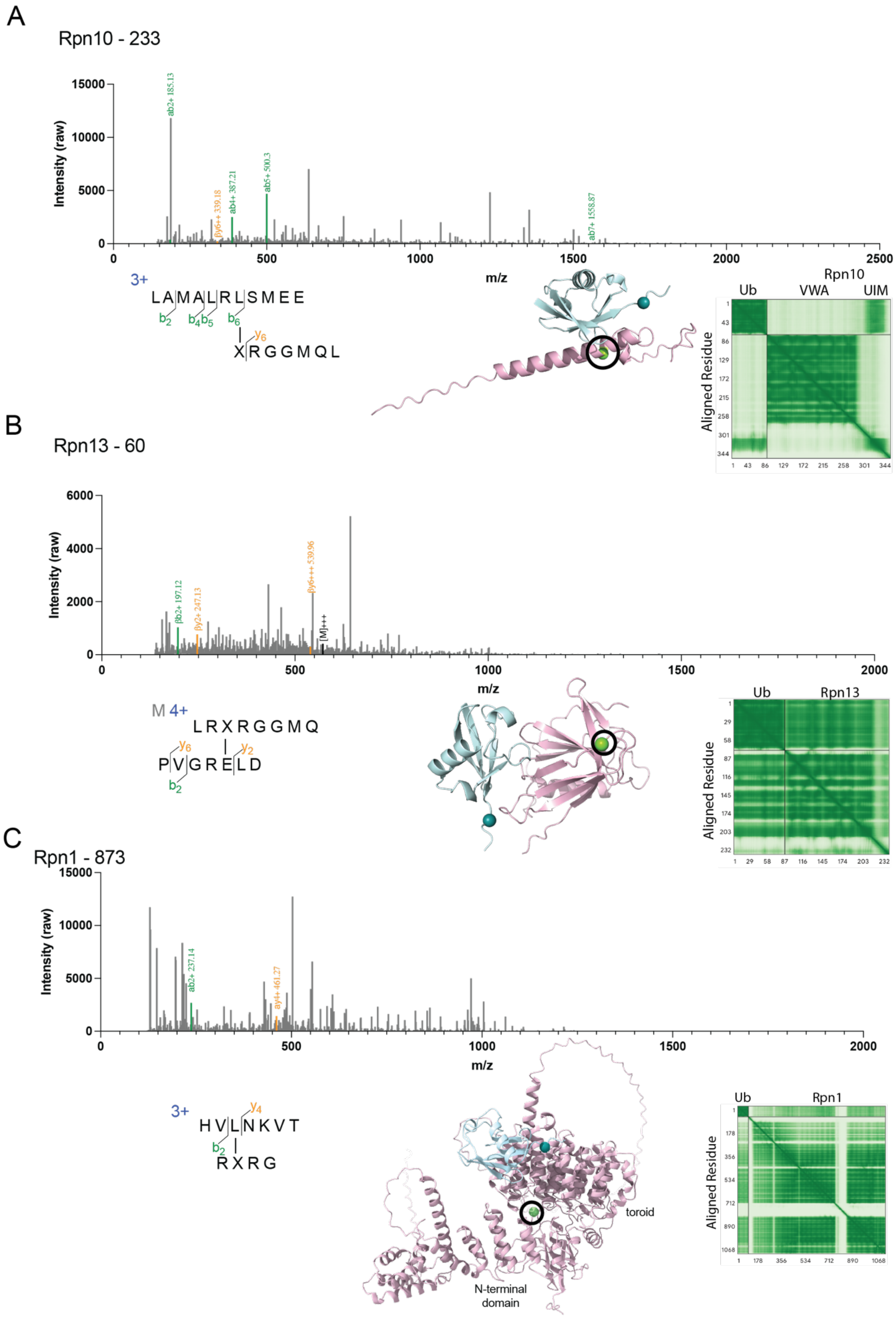
A
Rpn10 - 233
Intensity (raw)
m/z
ab2+ 185.13
βy6++ 339.18
ab4+ 387.21
ab5+ 500.3
ab7+ 1558.87
3+
LAMALRLSMEE
XRGGMQL
Rpn10
Ub
VWA
UIM
Aligned Residue
B
Rpn13 - 60
βb2+ 197.12
βy2+ 247.13
βy6+++ 539.96
[M]+++
M 4+
LRXRGGMQ
PVGRELD
Ub
Rpn13
Aligned Residue
C
Rpn1 - 873
ab2+ 237.14
αy4+ 461.27
3+
HVLNKVT
RXRG
toroid
N-terminal
domain
Ub
Rpn1
Aligned Residue

Supporting Figure S5: Ub(BPA)-receptor crosslink analyses. A) Mass spectrum of identified peptide containing a crosslink to position 233 in Rpn10 (top) and schematic of identified peptide (bottom left). AlphaFold structural model of ubiquitin bound to Rpn10 (bottom middle), with crosslink site in Rpn10 shown as a green sphere and the BPA in Ub(L73BPA) indicated as a teal sphere. The AlphaFold predicted error plot is show on the right, with higher confidence residues colored green and lower confidence residues colored white. **B)** Mass spectrum of identified peptide containing a crosslink to position 60 of Rpn13 (top) and schematic of identified peptide (bottom left). AlphaFold structural model of ubiquitin bound to Rpn13 (bottom middle), with crosslink site in Rpn13 shown as a green sphere and the BPA in Ub(L73BPA) indicated as a teal sphere. The AlphaFold predicted error plot is show on the right, with higher confidence residues colored green and lower confidence residues colored white. **C)** Mass spectrum of identified peptide containing a crosslink to position 873 of Rpn1 (top) and schematic of identified peptide (bottom left). AlphaFold structural model of ubiquitin bound to the toroid domain of Rpn1 (bottom middle), with crosslink site L873 in a cleft between Rpn1's toroid and N-terminal domain shown as a green sphere and the BPA position in Ub(L73BPA) shown as a teal sphere. The AlphaFold predicted error plot is shown on the right, with higher confidence residues colored green, and lower confidence residues colored white.

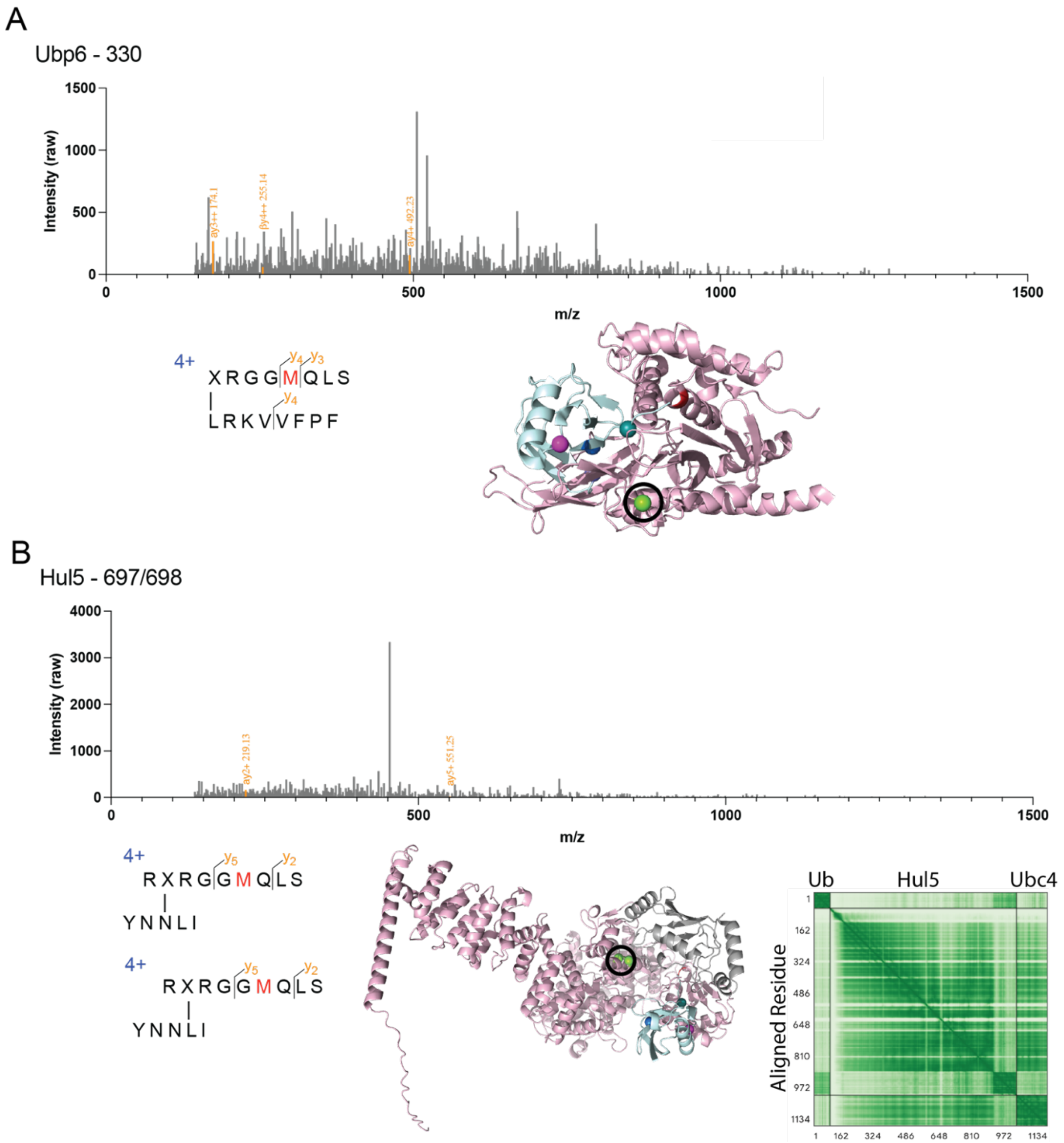

Supporting Figure S6: Crosslinks to proteasome-bound Ubp6 and Hul5. A) Mass spectrum of an identified peptide containing a crosslink at position 330 of Ubp6 (top) and schematic of identified peptide (bottom left). Structure of ubiquitin bound to Ubp6 (PDB: 7QO4), with crosslink site in Ubp6 shown as a green sphere and the BPA position in Ub(L73BPA) indicated as a teal sphere (bottom right). **B)** Mass spectrum of identified peptide containing a crosslink to positions 697/698 of Hul5 (top) and schematic of identified peptide (bottom left). AlphaFold structural model of ubiquitin bound to Hul5 (bottom middle), with crosslink site in Hul5 shown as a green sphere and the BPA in Ub(L73BPA) shown as a teal sphere. The AlphaFold predicted error plot is shown on the right, with higher confidence residues colored green and lower confidence residues colored white.

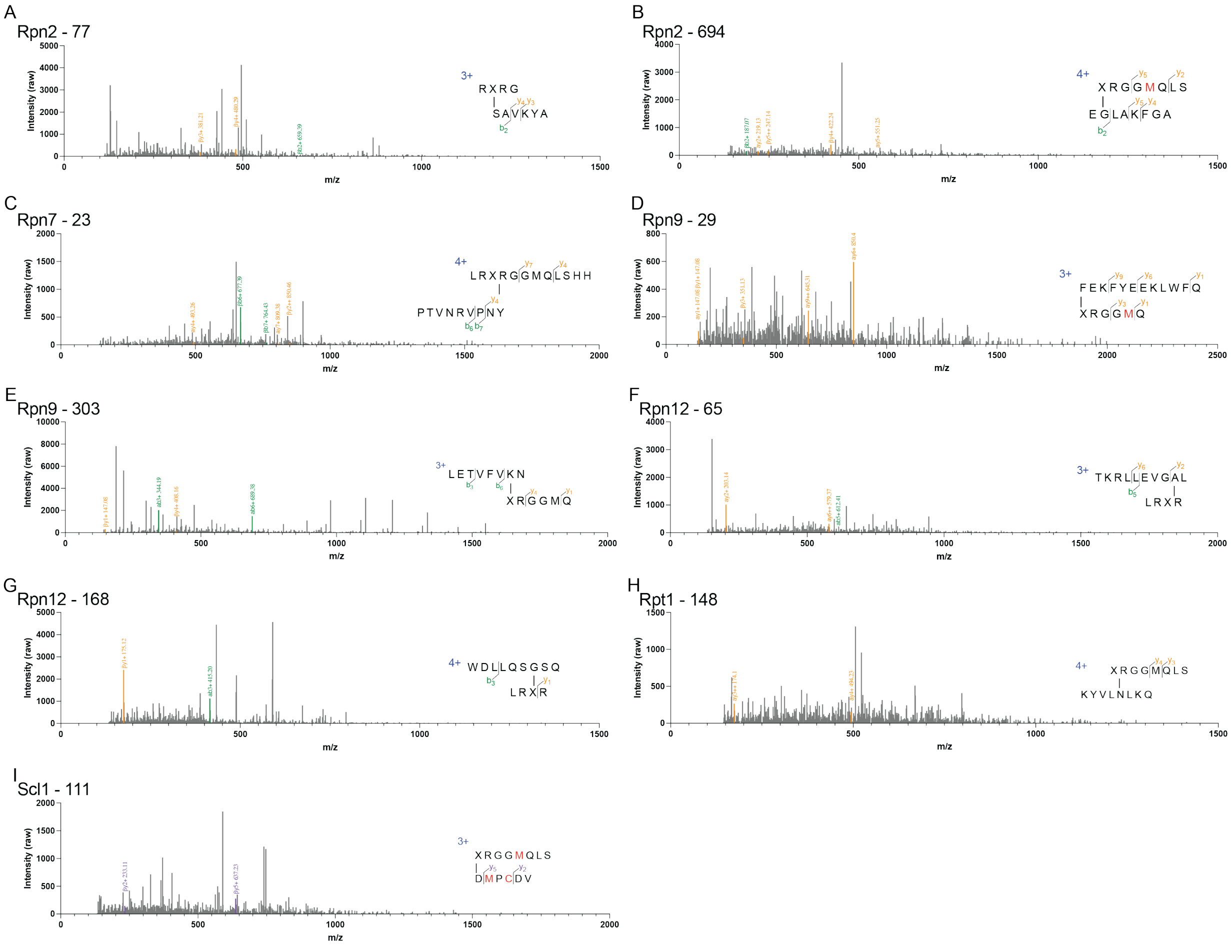

Supporting Figure S7: Mass spectra of crosslinked peptides from proteasomal subunits. A) Mass spectrum of an identified peptide containing a crosslink at position 77 in Rpn2 77 (left) and schematic of this peptide (right), with BPA indicated as 'X'. **B)** Mass spectrum of an identified peptide containing a crosslink at position 294 in Rpn2 (left) and schematic of this peptide (right), with BPA indicated as 'X'. **C)** Mass spectrum of an identified peptide containing a crosslink at position 23 in Rpn7 (left) and schematic of this peptide (right), with BPA indicated as 'X'. **D)** Mass spectrum of an identified peptide containing a crosslink at position 29 in Rpn9 (left) and schematic of this peptide (right), with BPA indicated as 'X'. **E)** Mass spectrum of an identified peptide containing a crosslink at position 303 of Rpn9 (left) and schematic of this peptide (right), with BPA indicated as 'X'. **F)** Mass spectrum of an identified peptide containing a crosslink at position 65 of Rpn12 (left) and schematic of this peptide (right), with BPA indicated as 'X'. **G)** Mass spectrum of an identified peptide containing a crosslink at position 168 of Rpn12 (left) and schematic of this peptide (right), with BPA indicated as 'X'. **H)** Mass spectrum of an identified peptide containing a crosslink at position 148 of Rpt1 (left) and schematic of this peptide (right), with BPA indicated as 'X'. **I)** Mass spectrum of an identified peptide containing a crosslink at position 111 of Scl1 (left) and schematic of identified peptide (right), with BPA indicated as 'X'.
